# Public Awareness About the Role of Anesthesiologists in Jeddah, Saudi Arabia: A Cross-Sectional Study

**DOI:** 10.7759/cureus.83245

**Published:** 2025-04-30

**Authors:** Abdurahman Bazaid, Amal Alrajhi, Jihan J Siddiqui, Samaa T Shoukry, Almaha Alshehri, Abdullah A AlDhuwaihy, Abdulrahman F Masoud, Nawaf S Alhazmi, Ali Alamri, Qasi Alabdali, Haifaa Kayal

**Affiliations:** 1 College of Medicine, King Saud Bin Abdulaziz University for Health Sciences, Jeddah, SAU; 2 College of Medicine, King Abdulaziz University, Jeddah, SAU; 3 Anesthesiology, International Medical Center Hospital, Jeddah, SAU; 4 Anesthesiology, Dr. Soliman Fakeeh Hospital, Jeddah, SAU; 5 College of Medicine, University of Jeddah, Jeddah, SAU; 6 College of Medicine, King Saud University, Riyadh, SAU; 7 Department of Anesthesiology, King Saud Bin Abdulaziz University for Health Sciences College of Medicine, Jeddah, SAU

**Keywords:** anesthesiologist, anesthesiology, patient education, public awareness, surgery

## Abstract

Background

Anesthesiologists play a critical role in patient care before, during, and after surgery. Despite their importance, public awareness of their roles remains low. This study aimed to assess public knowledge regarding anesthesiologists’ responsibilities in Jeddah, Saudi Arabia, focusing on preoperative, intraoperative, and postoperative phases.

Methodology

A cross-sectional, observational study was conducted from September 2023 to September 2024. Participants from Jeddah, Saudi Arabia, aged 18 years and above, were recruited via social media advertisements. A web-based questionnaire was used to gather data, adapted from a survey created by the Korean Society of Anesthesiologists. Descriptive statistics and chi-square tests were used for analysis, with a sample size of 449 respondents.

Results

The majority of participants (n = 369, 82.2%) correctly identified anesthesiologists as responsible for administering anesthesia during surgery. Additionally, 275 (61.2%) participants recognized that surgeons and anesthesiologists have distinct roles (p = 0.037). Nearly 252 (56.1%) participants believed anesthesiologists determine patient eligibility for surgery, while 225 (50.1%) acknowledged their role in controlling vital signs during surgery. Among those scheduled for admission, 306 (68.2%) preferred receiving detailed pre-surgical anesthesia information. Social media was the most preferred platform for raising awareness (n = 216, 48.1%). A significant gender difference was observed in perceptions of responsible persons for resuscitation, patient recovery, and blood transfusion during surgery (p < 0.001).

Conclusions

Public awareness of anesthesiologists’ roles remains limited, particularly outside the operating room. Targeted educational campaigns using digital platforms could improve understanding and reduce misconceptions about the role of anesthesiologists, enhancing patient satisfaction and recruitment into the field.

## Introduction

Anesthesiology is a medical specialty that focuses on the care of patients before, during, and after surgery. Anesthesiologists play a vital role in ensuring the safety and comfort of patients undergoing a wide range of procedures, from minor dental surgery to complex open-heart surgery by performing a full medical check-up before surgery to ensure patient safety, continuous and close monitoring of life-sustaining vital signs during surgery, and minimizing the level of pain after surgery. Despite their importance, public awareness about the specialty of anesthesiology and the role of anesthesiologists is often low. A 2014 survey found that only 25% of respondents knew that anesthesiologists oversaw anesthesia during surgery [1,2]. Additionally, many people believe that surgeons are responsible for monitoring a patient’s vital signs during surgery, when, in fact, this is one of the primary roles of the anesthesiologist [3]. There are several reasons why public awareness of anesthesiology may be low. One reason is that anesthesiologists work largely behind the scenes. During surgery, patients are typically unconscious and unaware of the anesthesiologist’s presence. Additionally, anesthesiologists often do not have the privilege to meet patients and their families for several visits to answer questions. Another reason for low public awareness of anesthesiology may be the misconception that anesthesia is simply putting a patient to sleep and waking them up again.

Anesthesia is a complex process that requires a deep understanding of physiology, pharmacology, and critical care medicine. Anesthesiologists must carefully monitor patients’ vital signs and adjust the anesthesia plan as needed to ensure their safety. In addition to their role in surgery, anesthesiologists also play a vital role in pain management and critical care medicine. Anesthesiologists work in pain clinics to help patients manage chronic pain, and they also provide care for patients in intensive care units [2]. Despite the importance of their work, anesthesiologists are often underappreciated, especially in Saudi Arabia. This is likely due to a lack of public awareness about their specialty and the role they play in patient care [4]. Moreover, the sample size used in Saudi research is not enough to make an accurate estimation of the public knowledge. On the other hand, a Canadian research study had a large sample size and reported an accurate estimation of the public perception [5].

It is important to raise public awareness about anesthesiology for several reasons. First, it can help reduce patient anxiety and improve patient satisfaction with care. When patients understand the role of the anesthesiologist and the safety measures that are in place, they are more likely to feel comfortable and confident going into surgery. Second, raising public awareness about anesthesiology can help attract more talented students to the field. Anesthesiology is a challenging but rewarding specialty, and it is important to ensure that there is a sufficient supply of qualified anesthesiologists to meet the needs of the population. Finally, raising public awareness about anesthesiologists by organizing campaigns and medical education can help promote advocacy for the specialty. Anesthesiologists play a vital role in the healthcare system, and they must have a strong voice in decision-making. By raising public awareness about anesthesiology, we can help ensure that anesthesiologists have the resources they need to provide the best possible care for their patients.

## Materials and methods

This observational, cross-sectional, questionnaire-based study was conducted via social media advertisements. From September 20, 2023, to September 20, 2024, Jeddah citizens answered the electronic survey regarding their knowledge about the role of anesthesiologists in Jeddah, Saudi Arabia. This study was conducted at King Abdulaziz Medical City (KAMC) and extended to the city-wide public in Jeddah, Saudi Arabia, by using web advertisements. KAMC is a 751-bed tertiary care center established in July 1982 in Jeddah by the National Guard Health Affairs to provide medical care services for the National Guard personnel and their dependents and the public in the western region. It has several complexes such as the Cardiology Center, Outpatient Clinic, Princess Norah Oncology Center, and the Bone Marrow Transplantation Center.

The study participants were adults aged more than 18 years or older living in Jeddah, Saudi Arabia. The convenience sampling technique was employed to recruit participants. The sample size was calculated using Raosoft software. Because the total population in Jeddah city during the study was expected to be more than 3.9 million, the required sample size was estimated at 95% confidence level with a margin of error of ±5% and a 50% rate of distribution. The required minimum size was determined to be 385 [6].

Citizens of Jeddah city were recruited through social media advertising using a web-based questionnaire via Google Forms. The participants were provided with the link to answer the survey. The survey was created by the Korean Society of Anesthesiologists and used by Lee et al. [1]. A consent form was needed as this study used electronic surveys for data collection. Participants’ privacy and confidentiality were assured, with all data, hard and soft.

Statistical analysis

Descriptive statistics were presented as frequencies and percentages for categorical variables. Numerical data were presented as the mean and standard deviation (SD). Categorical data were presented as the frequency and percentage, analyzed using the chi-square test or Fisher’s exact test. SPSS Software version 27 (IBM Corp., Armonk, NY, USA) was used for the statistical analysis. P-values <0.05 were considered statistically significant.

## Results

A total of 449 participants participated in this study. The mean age of the participants was 35.96 years. Most participants (n = 342, 76.2%) had a bachelor’s degree. A large proportion of the participants (n = 184, 41%) were employees, while the rest were unemployed (n = 129, 28.7%) and students (n = 107, 23.8%). The average income of the majority of the participants (n = 176, 39.2%) was between 5,000 and 10,000 SAR (Table 1).

**Table 1:** Participants’ demographics (N = 449).

		Male (n = 144)	Female (n = 305)	Total (N = 449)
Age	Mean ± SD	35.49 ± 14.38	36.19 ± 13.61	35.96 ± 13.85
Education level	Pre-college education	30 (20.83%)	50 (16.39%)	80 (17.8%)
	Bachelor’s degree	105 (72.92%)	237 (77.7%)	342 (76.2%)
	Postgraduate studies	9 (6.25%)	18 (5.9%)	27 (6%)
Profession	Student	38 (26.39%)	69 (22.62%)	107 (23.8%)
	Unemployed	24 (16.67%)	105 (34.43%)	129 (28.7%)
	Employee	68 (47.22%)	116 (38.03%)	184 (41%)
	Freelancer	14 (9.72%)	15 (4.92%)	29 (6.5%)
Average income	<5,000	11 (7.64%)	23 (7.54%)	34 (7.57%)
	5,000–10,000	49 (34.03%)	127 (41.64%)	176 (39.2%)
	11,000–15,000	15 (10.42%)	47 (15.41%)	62 (13.81%)
	≥20,000	35 (24.31%)	20 (6.56%)	55 (12.25%)
	Not applicable	34 (23.61%)	88 (28.85%)	122 (27.17%)

Concerning the relationship between the surgeon and the anesthesiologist during the operation, there was a statistically significant difference, with the majority thinking that every doctor has a different role (n = 275, 61.2%). Gender-wise, more females (n = 190, 62.3%) thought that every doctor has a different role than males (n = 85, 59.03%) (p = 0.037). No other significant gender differences were observed in responses to the remaining questions (Table 2).

**Table 2:** Participants’ awareness regarding the roles of medical staff pre and intraoperatively. ꭓ^2^: chi-square value; *: significant at p-values <0.05.

Question:		Male (n = 144)	Female (n = 305)	Total (N = 449)	ꭓ^2^	P-value
Who is responsible for anesthesia during surgical operations?	Anesthesiologist	112 (77.78%)	257 (84.26%)	369 (82.2%)	6.711	0.145
	Anesthesia technician	22 (15.28%)	38 (12.46%)	60 (13.4%)		
	Surgery doctor	5 (3.47%)	7 (2.3%)	12 (2.7%)		
	Nursing staff	2 (1.39%)	0 (0%)	2 (0.4%)		
	I don’t know	3 (2.08%)	3 (0.98%)	6 (1.3%)		
During the operation, what is the relationship between the surgeon and the anesthesiologist?	Every doctor has a different role	85 (59.03%)	190 (62.3%)	275 (61.2%)	8.487	0.037*
	Under an anesthesiologist’s orders	21 (14.58%)	50 (16.39%)	71 (15.8%)		
	Under the surgeon’s orders	32 (22.22%)	39 (12.79%)	71 (15.8%)		
	I don’t know	6 (4.17%)	26 (8.52%)	32 (7.1%)		
Who determines whether a patient qualifies for surgery?	Anesthesiologist	85 (59.03%)	167 (54.75%)	252 (56.1%)	2.729	0.435
	Surgery doctor	50 (34.72%)	117 (38.36%)	167 (37.2%)		
	Nursing staff	4 (2.78%)	4 (1.31%)	8 (1.8%)		
	I don’t know	5 (3.47%)	17 (5.57%)	22 (4.9%)		
Who decides whether a patient can eat before surgery?	Anesthesiologist	92 (63.89%)	181 (59.34%)	273 (60.8%)	2.504	0.475
	Surgery doctor	43 (29.86%)	92 (30.16%)	135 (30.1%)		
	Nursing staff	4 (2.78%)	11 (3.61%)	15 (3.3%)		
	I don’t know	5 (3.47%)	21 (6.89%)	26 (5.8%)		

Participants were also asked about the roles of each care provider intraoperatively. The question of who is responsible for blood transfusion during surgery showed a statistically significant difference (p < 0.001). The nursing staff was the most frequently reported as being responsible (n = 228, 50.8%), with a higher proportion of males (n = 85, 59.03%) compared to females (n = 143, 46.89%) (Table 3). Moreover, the staff responsible for resuscitating the patient during surgery showed a statistically significant difference (p < 0.001). Surgeons were the most frequently reported as responsible (n = 158, 35.2%), with a higher number of females (n = 128, 41.97%), compared to males (n = 30, 20.83%) (Table 3). No other significant gender differences were observed in responses to the remaining questions.

**Table 3:** Public’s awareness about the roles in the intraoperative period. ꭓ^2^: chi-square value; *: significant at p-values <0.05.

Question:		Male (n = 144)	Female (n = 305)	Total (N = 449)	ꭓ^2^	P-value
Who controls vital signs such as blood pressure and heart rate during surgery?	Anesthesiologist	70 (48.61%)	155 (50.82%)	225 (50.1%)	0.587	0.899
	Surgery doctor	10 (6.94%)	17 (5.57%)	27 (6%)		
	Nursing staff	56 (38.89%)	119 (39.02%)	175 (39%)		
	I don’t know	8 (5.56%)	14 (4.59%)	22 (4.9%)		
Who gives and administers anesthetics and fluids during surgery?	Anesthesiologist	105 (72.92%)	244 (80%)	349 (77.7%)	4.128	0.248
	Surgery doctor	11 (7.64%)	12 (3.93%)	23 (5.1%)		
	Nursing staff	24 (16.67%)	40 (13.11%)	64 (14.3%)		
	I don’t know	4 (2.78%)	9 (2.95%)	13 (2.9%)		
Who can estimate the amount of blood loss during surgery?	Anesthesiologist	32 (22.22%)	56 (18.36%)	88 (19.6%)	1.761	0.623
	Surgery doctor	75 (52.08%)	155 (50.82%)	230 (51.2%)		
	Nursing staff	24 (16.67%)	58 (19.02%)	82 (18.3%)		
	I don’t know	13 (9.03%)	36 (11.8%)	49 (10.9%)		
Who transfuses blood when needed during surgery?	Anesthesiologist	37 (25.69%)	47 (15.41%)	84 (18.7%)	25.925	<0.001*
	Surgery doctor	11 (7.64%)	77 (25.25%)	88 (19.6%)		
	Nursing staff	85 (59.03%)	143 (46.89%)	228 (50.8%)		
	I don’t know	11 (7.64%)	38 (12.46%)	49 (10.9%)		
Who resuscitates the patient during surgery?	Anesthesiologist	51 (35.42%)	70 (22.95%)	121 (26.9%)	24.499	<0.001*
	Surgery doctor	30 (20.83%)	128 (41.97%)	158 (35.2%)		
	Nursing staff	49 (34.03%)	67 (21.97%)	116 (25.8%)		
	I don’t know	14 (9.72%)	40 (13.11%)	54 (12%)		

Participants were also asked about the roles of each care provider postoperatively. The question of who is responsible for the patient’s safe recovery in the recovery room showed a statistically significant difference (p = 0.023). The nursing staff was the most frequently seen as responsible (n = 148, 33%), with a higher proportion of females (n = 112, 36.72%) compared to males (n = 36, 25%) (Table 4). No other significant gender differences were observed in responses to the remaining questions.

**Table 4:** Public’s awareness regarding the roles in the postoperative period. ꭓ^2^: chi-square value; *: significant at p-values <0.05.

		Male (n = 144)	Female (n = 305)	Total (N = 449)	ꭓ^2^	P-value
Who ensures the patient recovers smoothly after surgery?	Anesthesiologist	32 (22.22%)	46 (15.08%)	78 (17.4%)	5.190	0.158
	Surgery doctor	89 (61.81%)	191 (62.62%)	280 (62.4%)		
	Nursing staff	20 (13.89%)	55 (18.03%)	75 (16.7%)		
	I don’t know	3 (2.08%)	13 (4.26%)	16 (3.6%)		
In the recovery room, who controls postoperative pain?	Anesthesiologist	62 (43.06%)	117 (38.36%)	179 (39.9%)	2.214	0.529
	Surgery doctor	30 (20.83%)	56 (18.36%)	86 (19.2%)		
	Nursing staff	46 (31.94%)	114 (37.38%)	160 (35.6%)		
	I don’t know	6 (4.17%)	18 (5.9%)	24 (5.3%)		
In the recovery room, who is responsible for the patient’s safe recovery?	Anesthesiologist	56 (38.89%)	81 (26.56%)	137 (30.5%)	9.505	0.023*
	Surgery doctor	40 (27.78%)	80 (26.23%)	120 (26.7%)		
	Nursing staff	36 (25%)	112 (36.72%)	148 (33%)		
	I don’t know	12 (8.33%)	32 (10.49%)	44 (9.8%)		

When asked about the role of the anaesthesiologist, the most frequently reported role was providing anesthesia for surgical procedures in the operating room (n = 423, 94.2%), while caring for emergency patients in the emergency room was the least recognized role. Gender-wise, more females (n = 292, 95.74%) than males (n = 131, 90.97%) identified anesthesia for surgery as the primary role (p = 0.04). No other significant differences were observed (Table 5).

**Table 5:** Perspectives of participants regarding the role of anesthesiologists. ꭓ^2^: chi-square value; *: significant at p-values <0.05.

	Male (n = 144)	Female (n = 305)	Total (N = 449)	ꭓ^2^	P-value
Anesthesia for surgical procedures in the operating room	131 (90.97%)	292 (95.74%)	423 (94.2%)	4.072	0.044*
Local anesthesia for minor surgical operations in clinics	63 (43.75%)	110 (36.07%)	173 (38.5%)	2.439	0.118
Care of emergency patients in the emergency room	19 (13.19%)	24 (7.87%)	43 (9.6%)	3.204	0.073
Care of patients in the intensive care unit	28 (19.44%)	38 (12.46%)	66 (14.7%)	3.807	0.051
Resuscitation anywhere in the hospital and pain management in the pain clinic	46 (31.94%)	72 (23.61%)	118 (26.3%)	3.510	0.061

Participants were asked about their preferences regarding pre-surgical information provided by an anesthesiologist, with the majority preferring detailed information (n = 306, 68.2%). Gender-wise, there was no statistically significant difference between males and females in their preference for receiving pre-surgical anesthesia information (p > 0.05) (Table 6).

**Table 6:** Requests for pre-surgical information about anesthesia provided by anesthesiologists. ꭓ^2^: chi-square value; *: significant at p-values <0.05.

Question		Male (n = 144)	Female (n = 305)	Total (N = 449)	ꭓ^2^	P-value
You are going to undergo surgery. Would you like to have an anesthesia method explained by a doctor specialized in anesthesia?	Yes. I want detailed information	89 (61.81%)	217 (71.15%)	306 (68.2%)	4.326	0.228
	Yes, but less detailed information due to anxiety	43 (29.86%)	68 (22.3%)	111 (24.7%)		
	No	9 (6.25%)	13 (4.26%)	22 (4.9%)		
	I don’t know	3 (2.08%)	7 (2.3%)	10 (2.2%)		

The largest proportion of the cohort (n = 216, 48.1%) viewed social media programs as an effective way to raise awareness about anesthesiologists. Other preferred methods included seminars led by specialists (n = 93, 20.7%), hospital information brochures (n = 51, 11.4%), information from the Society of Anesthesiologists (n = 49, 10.9%), and television shows (n = 40, 8.9%).

## Discussion

This study aimed to evaluate the public’s understanding of anesthesiologists’ roles in Jeddah, Saudi Arabia, focusing on their responsibilities during preoperative, intraoperative, and postoperative phases in the emergency room and patient floors. Our findings indicate that a significant majority of participants (82.2%) correctly identified the anesthesiologist as the professional responsible for administering anesthesia during surgical operations. This aligns with previous studies conducted in Saudi Arabia, where a substantial proportion of the public recognized anesthesiologists’ role in anesthesia administration. For example, a study conducted in Jeddah found that 62.2% of participants correctly identified the anesthesiologist as the one administering anesthesia [3]. When participants were asked who determined whether a patient qualifies for surgery, 56.1% correctly identified the anesthesiologist. This finding is consistent with studies indicating that while many individuals recognize the anesthesiologist’s role in anesthesia administration, their involvement in preoperative assessments is less acknowledged. For instance, according to the same study in Jeddah, 53.4% of participants thought the surgeon was in charge of managing postoperative pain [3]. Regarding the decision on preoperative fasting, 60.8% of participants correctly attributed this responsibility to the anesthesiologist. This aligns with findings from the Qassim Province, which noted that knowledge about regional anesthesia’s complications, for example, has a favorable impact on anesthetic knowledge [2].

In our study, 50.1% of participants correctly identified anesthesiologists as responsible for controlling vital signs such as heart rate and blood pressure during surgery. This aligns with the standard medical practice where anesthesiologists monitor and manage these parameters to ensure patient stability [1]. Similarly, a large proportion (77.7%) correctly identified anesthesiologists as responsible for administering anesthetics and fluids during surgery. This is consistent with the study by Almutairi et al. (2023), which highlighted the integral role of anesthesiologists in intensive care units, operating rooms, and pain clinics [7]. Similarly, the question about resuscitation during surgery showed a significant gender difference (p < 0.001), with female participants more likely to attribute this responsibility to surgeons (41.97%) compared to male participants (20.83%). This contrasts with existing studies, where anesthesiologists are often recognized as key players in resuscitation during surgery, particularly in managing airway and respiratory issues [8]. However, this also aligns with a Jordanian study, where male surgeons were seen as more experienced and informed, while female surgeons were seen as more cooperative and compassionate [9].

Despite the critical role of anesthesiologists in postoperative care, public awareness remains limited. A national survey in Korea found that 25.2% of respondents were unaware of the role of anesthesiologists play both inside and outside the operating room, even though they are responsible for providing anesthesia during surgery [1]. Similarly, a study in Palestine reported that when it came to anesthesia and the functions of anesthesiologists, particularly outside of the operating room, perioperative patients lacked adequate knowledge. The study highlighted that patients’ awareness was higher regarding the preoperative and intraoperative roles of anesthesiologists but lower concerning their postoperative roles [10]. These results can be explained by the fact that patients frequently report feeling drowsy and having trouble recalling events that occurred or how they interacted with the anesthesiologists in the post-anesthesia care unit after surgery. Often, this is attributed to the impact of anesthesia and its subsequent aftermath on consciousness and knowledge of the anesthesiologists’ actions in the recovery room [10].

In this study, a significant proportion of participants correctly identified anesthesiologists as responsible for providing anesthesia during surgical procedures in the operating room (94.2%). This is in line with a study conducted in Romania, which found that 94.6% of respondents correctly identified anesthesiologists as responsible for providing anesthesia in the operating room. However, the study also noted a significant lack of awareness regarding other critical roles of anesthesiologists, such as managing postoperative pain and providing intensive care [11]. Similarly, a study in Nepal reported that while 70% of participants recognized anesthesiologists’ involvement in the postoperative period, their roles in the operating room, intensive care unit, and pain management were less well understood [12]. The question about caring for emergency patients in the emergency room revealed that 9.6% of participants identified anesthesiologists as responsible. This finding contrasts with the study by Marulasiddappa and Nethra (2016), which found that 44% of participants were unaware that anesthesiologists are doctors [13]. Our study observed significant gender differences in responses, particularly regarding the role of anesthesiologists in providing anesthesia for surgical procedures. This finding is consistent with research indicating that women are more likely to perceive and experience gender-based discrimination and mistreatment in the medical field, including in specialties such as anesthesiology [14].

Despite the high recognition of anesthesiologists’ role in the operating room, there is a notable lack of awareness regarding their involvement in other critical areas such as emergency care, intensive care units, and pain management clinics. The American Society of Anesthesiologists emphasizes that patient care during the whole surgical procedure, i.e., before, during, and after the actual surgery procedure, is the responsibility of anesthesiologists [8]. Enhancing public education about the diverse roles of anesthesiologists is essential. Efforts to increase media exposure and strengthen the anesthesiologist-patient relationship can help improve the public’s understanding of the specialty and its practitioners [15,16].

A substantial proportion of participants expressed a preference for receiving detailed information about anesthesia approaches before surgery. This is in line with results from a survey of surgical patients’ viewpoints, which showed that 28% of respondents advocated collaborative decision-making with their clinical team and 43% chose a patient-led role. However, just 7.8% of respondents mentioned they participated fully in the decision-making process regarding the anesthetic they were given [17]. Our study found no statistically significant gender differences in the preference for receiving detailed pre-surgical anesthesia information (p = 0.228). This contrasts with a study on the influence of gender and anesthesia type on day surgery anxiety, which found that female patients were statistically significantly more anxious and desired more information compared to male patients [18]. Furthermore, a study on anesthesiologist-to-patient communication noted that female gender was one of the risk variables most strongly associated with decreased satisfaction and perioperative anxiety [19].

Our findings indicate a strong inclination toward utilizing social media platforms, with 48.1% of participants favoring this approach. Other preferred methods included seminars led by specialists (20.7%), hospital information brochures (11.4%), information from the Society of Anesthesiologists (10.9%), and television shows (8.9%). The predominant preference for social media programs aligns with global trends in health communication. A study by Cohen et al. highlighted that smart marketing strategies, including the use of social media, can significantly enhance public understanding of medical professions, including anesthesiology [20]. The preference for social media as a source of health information is consistent with findings from a national survey in Korea. The study reported that television programs were the most common source of information about anesthesiologists, followed by the internet and informational brochures. This suggests that the public is increasingly turning to digital platforms for health-related information [1].

Study strengths and limitations

This study has several strengths, including a large sample size derived from a population of over 3.9 million citizens in Jeddah, Saudi Arabia, which provides a robust estimate of public awareness about anesthesiology. The use of an electronic survey allowed for widespread accessibility and efficient data collection through social media advertisements, making it an effective method for reaching a diverse demographic. Additionally, the study employed a well-established questionnaire validated by the Korean Society of Anesthesiologists. However, there are limitations related to the convenience sampling technique, which may introduce selection bias, as tech-savvy participants may have more exposure to the study. The self-reported nature of the survey also means that responses are susceptible to recall and social desirability biases. Furthermore, the cross-sectional design only provides a snapshot of public awareness, and the findings may not reflect trends over time.

The findings from this study could have important implications for future educational initiatives aimed at improving public awareness of anesthesiologists’ roles, especially in Saudi Arabia. By identifying gaps in public knowledge, targeted campaigns and educational programs can be designed to address misconceptions, such as the role of anesthesiologists in areas beyond surgery, including critical care and pain management.

## Conclusions

This study highlights significant gaps in public awareness regarding the role of anesthesiologists, especially outside the operating room. While most participants recognized anesthesiologists’ role in administering anesthesia during surgery, their involvement in critical care, pain management, and emergencies was less acknowledged. Gender differences were observed, particularly in perceptions of who is responsible for resuscitation during surgery. A strong preference for social media programs to raise awareness was noted, aligning with global trends in health communication. Efforts should focus on enhancing public education through digital platforms and medical education campaigns, emphasizing the multifaceted roles of anesthesiologists. Increasing awareness can help reduce patient anxiety, improve satisfaction, and attract more talent to the field, ensuring a robust future workforce for anesthesiology in Saudi Arabia.

## Appendices

Study questionnaire

Demographics

A. Age

B. Gender

C. Education

D. Occupation

The Public’s Awareness Regarding the Roles of Those Involved in Surgery with Anesthesia and the Roles in the Preoperative Period

1. In your opinion, who is in charge of anesthesia for surgery?

a. Technician

b. Surgeon

c. Doctor specialized in anesthesia

d. Nurse

e. Don’t know

2. During an operation, what is the relationship between the surgeon and the anesthesiologist?

a. Under the surgeon’s orders

b. Under the anesthetic doctor’s orders

c. Each has different roles

d. Don’t know

3. Who determines whether the patient is fit for surgery (operability)?

a. Surgeon

b. Doctor specialized in anesthesia

c. Nurse

d. Don’t know

4. Who decides if a patient can eat before surgery?

a. Surgeon

b. Doctor specialized in anesthesia

c. Nurse

d. Don’t know

The Public’s Awareness Regarding the Roles in the Intraoperative Period

1. Who controls vital signs such as blood pressure and heart rate during an operation?

a. Surgeon

b. Doctor specialized in anesthesia

c. Nurse

d. Don’t know

2. Who administers the anesthetic drugs and fluids during an operation?

a. Surgeon

b. Doctor specialized in anesthesia

c. Nurse

d. Don’t know

3. Who estimates blood loss during an operation?

a. Surgeon

b. Doctor specialized in anesthesia

c. Nurse

d. Don’t know

4. Who transfuses blood when needed during an operation?

a. Surgeon

b. Doctor specialized in anesthesia

c. Nurse

d. Don’t know

5. Who resuscitates the patient during an operation?

a. Surgeon

b. Doctor specialized in anesthesia

c. Nurse

d. Don’t know

The Public’s Awareness Regarding the Roles in the Postoperative Period

1. Who makes sure the patient recovers smoothly after surgery?

a. Surgeon

b. Doctor specialized in anesthesia

c. Nurse

d. Don’t know

2. In the recovery room, who manages the postoperative pain?

a. Surgeon

b. Doctor specialized in anesthesia

c. Nurse

d. Don’t know

3. In the recovery room, who is responsible for the patient’s safe recovery?

a. Surgeon

b. Doctor specialized in anesthesia

c. Nurse

d. Don’t know

In the Hospital, What is the Role of a Doctor Specialized in Anesthesia (Select What You Believe)

a. Anesthesia for surgery in the operating room

b. Emergency patient care in the emergency room

c. Patients’ care in the intensive care unit

d. Local anesthesia for simple surgery in the OPD

e. Resuscitation anywhere in the hospital

f. Pain management in the pain clinic

Public Requests for Pre-surgical Information About Anesthesia Provided by a Doctor Specializing in Anesthesia

1. If you are to undergo an operation, do you want to receive an explanation of anesthesia from a doctor specialized in anesthesia?

a. Yes. I want detailed information.

b. Yes, but less detailed information due to anxiety

c. No.

d. I don’t know.

The Public’s Experience of the Role of a Doctor Specialized in Anesthesia During Surgery in the Mass Media, and Their Opinion About How to Raise Awareness About Doctors Specialized in Anesthesia

a. Television program

b. d. e. Informational brochure in the hospital

c. Information via the internet

d. Seminar by a doctor specialized in anesthesia

e. Information from the society of anesthesiologists
